# Migration and Fate of Acid Mine Drainage Pollutants in Calcareous Soil

**DOI:** 10.3390/ijerph15081759

**Published:** 2018-08-16

**Authors:** Fenwu Liu, Xingxing Qiao, Lixiang Zhou, Jian Zhang

**Affiliations:** 1 Environmental Engineering Laboratory, College of Resource and Environment, Shanxi Agricultural University, Taigu 030801, China; qiaoxingxing@stu.sxau.edu.cn (X.Q.); zhangjian@sxau.edu.cn (J.Z.); 2 Department of Environmental Engineering, College of Resources and Environmental Sciences, Nanjing Agricultural University, Nanjing 210095, China; lxzhou@njau.edu.cn

**Keywords:** acid mine drainage, Fe, S, calcareous soil, pollutant migration behavior

## Abstract

As a major province of mineral resources in China, Shanxi currently has 6000 mines of various types, and acid mine drainage (AMD) is a major pollutant from the mining industry. Calcareous soil is dominant in western North China (including the Shanxi Province), therefore, clarifying the migration behavior of the main AMD pollutants (H^+^, S, Fe, heavy metals) in calcareous soil is essential for remediating AMD-contaminated soil in North China. In this study, the migration behavior of the main pollutants from AMD in calcareous soil was investigated using soil columns containing 20 cm of surficial soil to which different volumes of simulated AMD were added in 20 applications. Filtrate that was discharged from the soil columns and the soil samples from the columns were analyzed. Almost all of the Fe ions (>99%) from the AMD were intercepted in the 0–20 cm depth of the soil. Although >80% of SO_4_^2−^ was retained, the retention efficiency of the soil for SO_4_^2−^ was lower than it was for Fe. Cu, as a representative of heavy metals that are contained in AMD, was nearly totally retained by the calcareous soil. However, Cu had a tendency to migrate downward with the gradual acidification of the upper soil. In addition, CaCO_3_ was transformed into CaSO_4_ in AMD-contaminated soil. The outcomes of this study are valuable for understanding the pollution of calcareous soil by AMD and can provide key parameters for remediating AMD-contaminated soil.

## 1. Introduction

In China, the mining industry is an important pillar industry that provides essential energy and other resources for national economic development [1]. There were more than 63,433 metal and non-metal mines in China in 2015, and the quantity of non-metal mines exceeds 56,600. What is more, the number of coal mines in China exceeds 15,000 [2]. As a major province of mineral resources in China, Shanxi currently (2018) has 6000 mines of different types. Notably, the rapid development of the coal mining industry in the Shanxi Province has had great effects on the province’s economic development [3]. However, mining activity is a double-edged sword for society. On the one hand, the industry produces economic benefits, however on the other hand, it causes serious contamination to the environment [4]. The environmental issues around mining areas are primarily related to mining-related surface disturbance [5], tailings waste pile production [6], dust pollution [7], and acid mine drainage (AMD) [8].

The oxidation of sulfide minerals that are associated with metal ore and coal ore is the main cause of AMD from mining activity [9]. Pyrite (FeS_2_) is the most common sulfide mineral that is responsible for the occurrence of AMD [10]. Therefore, AMD is very acidic (pH < 3.0) and normally contains a high amount of iron, sulfate, and a certain amount of heavy metals [11,12]. Inadequately treated AMD is a very widespread environmental problem that directly affects the healthy development of the mining industry [13]. Many researchers have studied environmental problems in soil and aquatic ecosystems that have arisen from acid mine drainage in China and elsewhere [5,14,15,16,17,18]. In fact, soil that has been polluted by iron and sulfate has attracted extensive attention around the world. For example, iron and sulfate transformations in acid sulfate soils is a hot issue in the environmental field in Australia [19,20].

China is the most populous country in the world. By the end of 2015, China’s population reached 1.37 billion [21], accounting for 18.7% of the total world population. In addition, China is a traditionally agricultural country [22]. Therefore, the issue of cultivated land quality has always been very topical in China [23]. Unfortunately, in China, some farmland that is surrounded by mining areas has been severely polluted by AMD [3,5,14,24]. Therefore, it is important to accurately identify the migration behavior of AMD pollutants in Chinese soils.

In fact, many studies in the last decade have focused on the migration behavior of AMD pollutants in red soil with a pH < 7.00, located in South China [24,25,26]. Li et al. [25] found that in the 0–15 cm surface horizon soils that were collected from the Dabaoshan Mountain of the Guangdong Province in South China, the total contents of Cu, Pb, Zn, and Cd were significantly higher in AMD-polluted soils by 17 times, 7 times, 5 times, and 2.5 times, respectively, compared to the unpolluted soils. Yang et al. [26] noted that a significant amount of SO_4_^2−^ was adsorbed by Fe/Al oxides and fine clays, and that a large amount of Fe existed as amorphous Fe oxide at the 20–30 cm mid-depth layer in AMD-contaminated soils along the Hengshi River of the Dabaoshan sulfide mining area, South China. In addition, Wang et al. [24] reported that AMD irrigation changed the composition and the diversity of the bacterial community in a paddy soil located in the Guangdong Province, and increased the abundance of sulfate-reducing bacteria in the soil.

Shanxi, a province in the northern part of China, is rich in mineral resources, especially coal resources [3]. In addition, the production of AMD as a result of mining activity in the Shanxi Province has been reported by a large number of researchers. Zhao et al. [27] investigated the geochemical characteristics of rare earth elements in AMD from the Sitai coal mine in the Shanxi Province and found that the sulfate complexes and free metal species in the AMD were dominant rare earth element species. Gao et al. [28] isolated an iron-oxidizing bacterium from AMD that was produced by the Zhongtiaoshan copper mine in the Shanxi Province. Notably, the dominant soil type in western North China, including the Shanxi Province, is calcareous with a high proportion of calcium carbonate (CaCO_3_) and a pH level often exceeding 8.0 [28,29,30]. Thus, the soil in North China has totally different characteristics from the red soil in South China (including the Guangdong Province). Unfortunately, the migration behavior of AMD pollutants in calcareous soil has never been reported. The clarification of this scientific problem is of great theoretical and practical significance for understanding the migration behavior of AMD pollutants in soil in northern China. Studying the migration of AMD pollutants in calcareous soil can help close up the data gap regarding soil pollution by AMD in China, especially in North China.

In view of this, the main objective of this study was to explore the migration behavior of key AMD pollutants (H^+^, Fe^3+^, SO_4_^2−^, and heavy metal ions, with Cu as an example) in calcareous soils using experimental soil columns.

## 2. Materials and Methods

### 2.1. Preparation of Simulated Acid Mine Drainage

Simulated AMD for the small-scale soil column experiment was prepared in 20 batch applications as follows. First, 15 mL of *A. ferrooxidans* LX5 inoculum [2] was added into each of a series of 250-mL Erlenmeyer flasks, each containing 50 mL of modified 9 K liquid medium stock solution and 85 mL of deionized water. The inorganic salt concentrations of the modified 9 K liquid medium (comprised of 0.0168 g of Ca(NO_3_)_2_, 0.058 g of K_2_HPO_4_, 0.119 g of KCl, 0.583 g of MgSO_4_·7H_2_O, 3.5 g of (NH_4_)_2_SO_4_, 44.2 g of FeSO_4_·7H_2_O in 1 L of deionized water, adjusted to pH 2.50) were increased three times in the stock solution. Then, the mixtures in the flasks were adjusted to pH ~2.50 and were incubated at 18–28 °C while being shaken at 150–180 rpm for 3–7 days until the ferrous ions were completely bio-oxidized. After incubation, the solution in each flask was filtered through quantitative filter paper to remove iron-based precipitate. The filtrate that was obtained in this process was considered to be the simulated AMD and was immediately stored at 4 °C for 2–3 days until use. During this period, the content of Fe, SO_4_^2−^, and the other measured elements had not been changed before or after being stored. Then, 0.0977 g of CuSO_4_·5H_2_O was dissolved in the 500 mL of simulated AMD to yield Cu^2+^ concentration ~50 mg/L. The pH, total Fe concentration, and SO_4_^2−^ concentration varied among the batch applications of the simulated AMD because the culture condition and the water evaporation degree were different during the preparation process. The pH of the simulated AMD for the soil column experiment varied from 2.22 to 2.49, the total Fe concentration varied from 7464.61 mg/L to 9328.02 mg/L, and the SO_4_^2−^ concentration varied from 19,542.25 mg/L to 35,035.21 mg/L.

### 2.2. Soil Column Experiment

Calcareous brown soil was collected from a site (112°34′28″ E, 37°25′30″ N) in Shanxi Agricultural University, Taigu, Shanxi Province, China. Ten subsamples were collected within 25 m^2^ using the plum blossom method from the surface soil layer (0–20 cm) in the sampling plot and were combined into a composite soil sample (~10 kg). The moisture content in situ soil was 11.64%, which can be calculated through the difference between the weight of the original collected soil and the weight of the 105 °C-dried soil. Some soil was air-dried and was passed through a 1-mm sieve to generate the samples for pH determination. Some soil was dried at 105 °C, was passed through a 0.15-mm sieve, and was analyzed for Fe, S, Cu, and Ca content. These analyses showed that the pH of the selected soil was 8.24, and the weight percentages of Fe, S, Cu, and Ca were 2.99%, 0.0337%, 0.0029%, and 5.35%, respectively. In addition, the clay fraction content in the tested soil was 19.9%.

**Figure 1:**
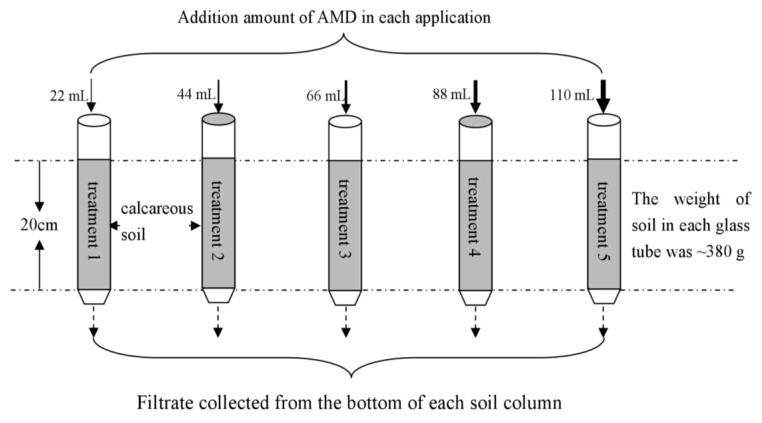
Schematic diagram of reaction columns (total of 20 batches of applications of AMD were added to each soil column over 132 days).

Fifteen glass columns (with a 25 cm length and 4.7 cm internal diameter) were uniformly packed to a depth of 20 cm with collected soil. The weight and density of the soil in each glass tube was ~380 g and ~1.042 g/cm^3^. Four layers of gauze were placed in the bottom of each soil column to prevent the loss of soil particles during the experiment. The pre-experiment results showed that this gauze application had no impact on the efficiency of the contaminant removal. Cu is one of the most important heavy metals in acid mine drainage in the Shanxi area. Therefore, the migration behavior of Cu from AMD in the calcareous soil was investigated in this study. Five treatments were conducted. The 22 mL (for treatment 1), 44 mL (for treatment 2), 66 mL (for treatment 3), 88 mL (for treatment 4), and 110 mL (for treatment 5) of the simulated AMD were added to the upper end of the soil columns during each application. The AMD properties that were applied to treatments 1 to 5 were consistent for the same application. All of the treatments were designed with three replicates. In fact, our team used the soil column (with a 20 cm effective length and 16 cm internal diameter) to carry out the related research pre-experiment with the addition of 0.25 L of stimulated AMD before carrying out this study. From the pre-experiment results, it was found that the AMD pollutants (such as Fe, SO_4_^2−^, Cu) were mainly intercepted in the soil. Therefore, on the basis of the pre-experiment results, this study sought to further investigate the migration of AMD pollutants in the surface 0–20 cm soil. The parameter of 22 mL of stimulated AMD came from the relevant parameters in the pre-experiment. Based on the selected 22 mL, the 44 mL, 66 mL, 88 mL, and 110 mL in this study were set according to equal difference. A total of 20 batches of applications of AMD were added to each soil column over 132 days. A schematic diagram of the reaction columns is shown in Figure 1. All of the soil columns were sealed and stored at 4 °C during days 78–108 because of the winter vacation. After each application of AMD, the pH of the filtrate that was collected from the bottom of each soil column (if any) was measured. In addition, 1 mL of filtrate was passed through a 0.22-μm membrane filter and the total Fe and SO_4_^2−^ concentrations in the filtrate were analyzed.

Among the 20 batches of applications of AMD, six batch applications (on days 25, 39, 58, 72, 122, and 132) were randomly selected for the analysis of Cu^2+^ concentrations in the filtrate. The Cu^2+^ removal efficiency of the soil was calculated based on the difference between the Cu^2+^ concentrations in the filtrate and in the simulated AMD as applied. When all 20 batches of the AMD applications had been completed, the soil columns in treatments 1–5 were separated at the middle of the column and were marked as “soil_0__–10cm_” and “soil_10__–20cm_”. A series of pH analyses were conducted on the airdried soil from the two groups of the samples that were passed through a 1-mm sieve. In this study, the AMD addition amount was the minimum value (22 mL for each application) in treatment 1 and the maximum value in treatment 5 (110 mL for each application). Soil samples from treatment 1 and treatment 5 were dried at 105 °C and were passed through a 0.15-mm sieve for analyses to determine the concentrations of Fe, S, Cu, and Ca, as well as soil morphology and soil mineralogy.

### 2.3. Analytical Procedures

The solution pH was measured using a pHS-3C model digital pH-meter [2] (Shanghai Yueping Scientific Instruments Co., Ltd., Shanghai, China). The total Fe concentration in the solution was determined using the 1, 10-phenanthroline method [31]. The SO_4_^2−^ concentration was determined using the barium sulfate turbidimetric method [32]. The Cu^2+^ concentration in the solution was determined using an atomic absorption spectrophotometer [33] (6810, Shanghai Senpu Technology Co., Ltd., Shanghai, China). The Cu^2+^ retention efficiency of a soil column was calculated as: Cu^2+^ retention efficiency (%) = [(C_0_ − C*_t_*)/C_0_] × 100% (where C*_0_* is the initial Cu^2+^ concentration and C*_t_* is the Cu^2+^ content in the filtrate from the soil columns in each application). The mineral phase or the morphology of the soil was determined by power X-ray diffraction (XRD) (MiniFlex II, Tokyo, Japan) using CuKα radiation (30 KV, 15 mA) or field-emission scanning electron microscopy (SEM) (JSM-7001F, Tokyo, Japan) [34]. The Fe and Ca contents in the soil were determined by X-ray fluorescence spectrometry (ZSX Primus II, Rigaku, Japan) [35]. The S content in the soil was determined using the combustion iodometric titration method [36]. In brief, the soil sample was placed in a tubular electric furnace at 1235–1300 °C, and the S element was converted to sulfur dioxide. Then, sulfur dioxide was absorbed by the distilled water and was titrated with an iodine standard solution. During the titration process, starch was used as an indicator.

### 2.4. Statistical Analysis

Data analysis was performed using Microsoft Excel^®^ 2010 (Microsoft Corporation, Redmond, WA, USA). All of the data points that are given in figures are mean values with their standard deviations to show their repeatability and reliability. All of the figures were drawn using Origin^®^ 7.5 software (OriginLab, Northampton, MA, USA).

## 3. Results and Discussion

### 3.1. Acid Buffering Performance of Calcareous Soil against AMD Pollution

**Figure 2:**
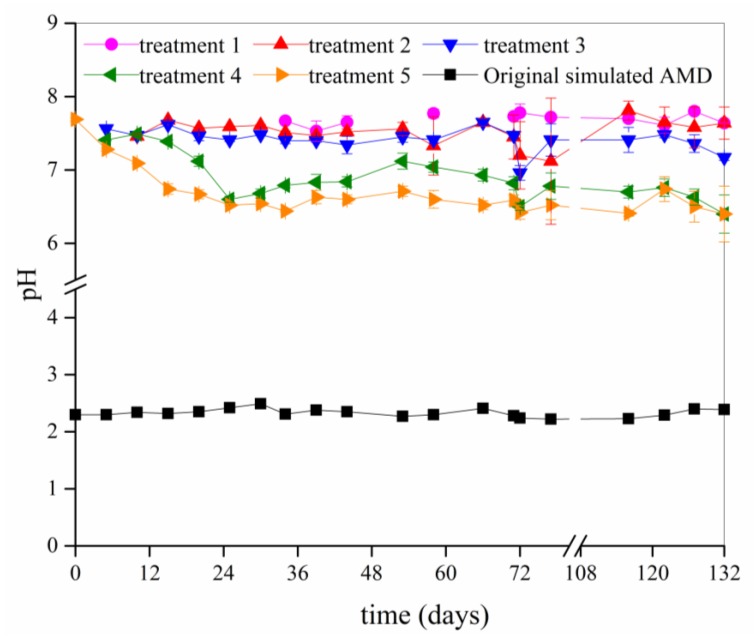
The pH of the filtrate that was collected from the bottom of the soil columns in the different treatments. (Treatments 1, 2, 3, 4, and 5 represent the addition of 22, 44, 66, 88, and 110 mL, respectively, of simulated acid mine drainage (AMD) to the soil columns at each application).

The acid buffering performance of calcareous soil against AMD pollution was evaluated by analyzing the changes in the pH of the filtrate that was collected from the bottom of the different soil columns (Figure 2).

In general, the maximum water-holding capacity for the calcareous brown soil in the Shanxi province ranges from 20% to 30% [37]. In this study, the initial soil moisture content (11.64%) was below the soil’s maximum water-holding capacity. The filtrate volume that was collected from the calcareous soil column in treatment 1 (22 mL AMD per application) was <5 mL during the first 30 days because the soil moisture content during this time did not reach the soil’s maximum water-holding capacity. The pH value of the filtrate could not be determined in this volume condition. Furthermore, over 30 mL (the pH value, total Fe and the SO_4_^2−^ of the filtrate can be determined in this volume condition) of the filtrate could be collected from the bottom of the soil columns in treatment 2 on day 10 (3rd batch application, 44 mL AMD/application), from the columns in treatment 3 on day 5 (2nd batch application, 66 mL AMD/application), from the columns in treatment 4 on day 5 (2nd batch application, 88 mL AMD/application), and from the columns in treatment 5 on day 0 (1st batch application, 110 mL AMD/application).

Although the calcareous soil became gradually acidified from the addition of AMD, the soil showed a strong acid buffering ability. For example, the pH of the filtrate that was collected from the bottom of the soil columns in treatments 1, 2, 3, 4, and 5 at 132 days were 7.64, 7.64, 7.17, 6.40, and 6.40, respectively. In short, during the entire experiment, 20 batches of the applications of the simulated AMD (with pH 2.22–2.49) totaling 440 mL (treatment 1) to 2200 mL (treatment 5) were added to ~380 g of calcareous soil, yet the pH of the filtrate that was collected from the columns only decreased from 7.81 to 6.40. In fact, in a previous study, we confirmed that the pH of the filtrate from the calcareous soil that was used in this study was close to the initial pH of AMD when the amount of AMD added was 10 times the quantity of the soil. During the current experiment, it was observed that a large amount of bubbles were generated at the surface of each soil column when AMD was added. This phenomenon may be attributed to the chemical reaction between calcium carbonate (CaCO_3_) in the calcareous brown soil and the H^+^ and SO_4_^2−^ in the simulated AMD, releasing carbon dioxide (CO_2_) according to the following reaction:

CaCO_3_ + 2H^+^ + SO_4_^2−^ → CaSO_4_ + CO_2_ + H_2_O (1)

### 3.2. Retention Efficiency of the Calcareous Soil for Total Fe, SO_4_^2−^, and Cu^2+^ Ions during AMD Application

**Figure 3:**
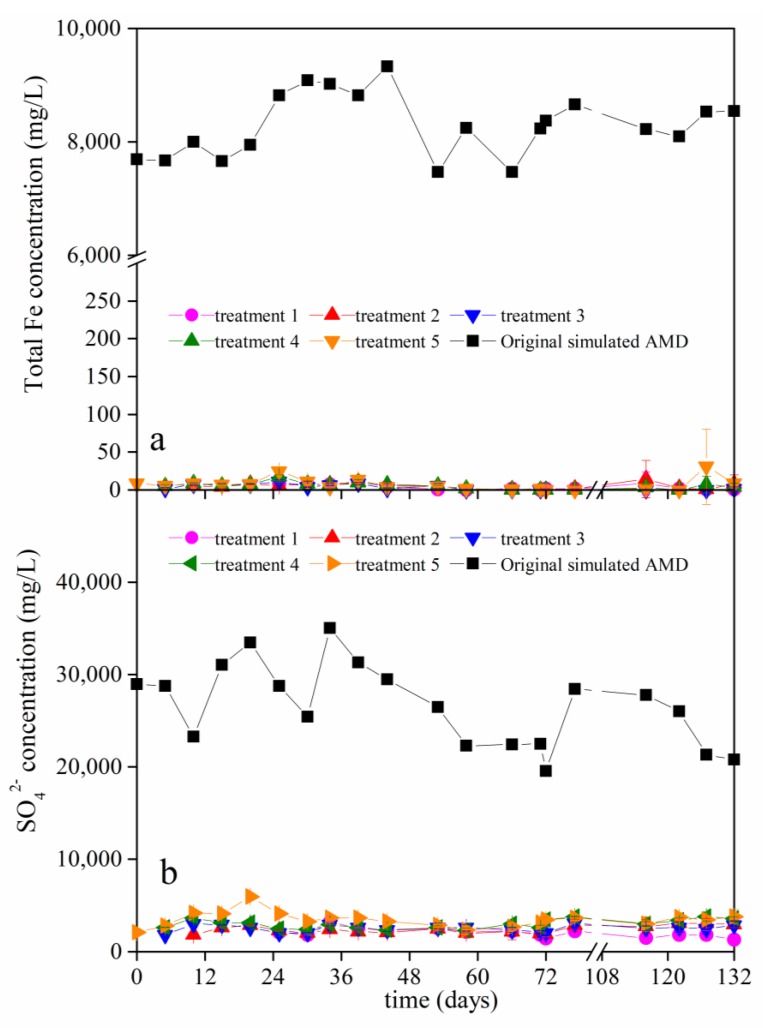
The concentrations of the (**a**) total Fe ions and (**b**) SO_4_^2−^ in the filtrate that was collected from the bottom of the soil columns in the different treatments. (Treatments 1, 2, 3, 4, and 5 represent the addition of 22, 44, 66, 88, and 110 mL, respectively, of simulated acid mine drainage (AMD) that was added to the soil columns at each application).

To better evaluate the migration behavior of Fe ions and SO_4_^2−^ ions in calcareous brown soil resulting from the AMD batch applications, the concentrations of the total Fe and SO_4_^2−^ ions in the filtrate that was collected from the bottom of the different soil columns were determined (Figure 3).

The initial concentration of total Fe ions in the different batches of AMD ranged from 7464.61 mg/L to 9328.02 mg/L. As can be seen in Figure 3a, the amount of total Fe ions in the filtrate that was collected from the bottom of different soil columns was in the range of 0–30.92 mg/L. In other words, the total Fe ions in AMD were nearly completely removed when the simulated AMD migrated vertically through 20 cm of calcareous brown soil. For example, in the 20th batch application, the retention efficiency for total Fe ions reached 99.99%, 99.91%, 99.98%, 99.95%, and 99.90% when 22 mL, 44 mL, 66 mL, 88 mL, and 110 mL, respectively, of AMD (pH 2.39, total Fe ions concentration 8542.06 mg/L) was added to the different soil columns. The efficient retention of total Fe in calcareous soil may mainly be due to Fe precipitation when the soil was polluted by AMD. According to Brittons [38], Fe^3+^ hydroxide precipitates at pH 3. During the system, pH changes from ~3 to ~7, Fe^3+^ may precipitate in the form of Fe(OH_3_), schwertmannite, or ferrihydrite, etc. [39,40]. Qiao et al. [32] reported that the total Fe removal efficiency reached 99.8% when AMD (pH 1.44, total Fe concentration ~3000 mg/L) was neutralized by CaO to pH ~7.

The initial concentrations of SO_4_^2−^ ions in the different batches of simulated AMD ranged from 19,542.25 mg/L to 35,035.21 mg/L. Figure 3b shows the concentration of SO_4_^2−^ ions in the filtrate that was collected from the different soil columns. For treatments 1, 2, 3, 4, and 5, the SO_4_^2−^ ion retention efficiency reached 90.08–94.67%, 85.43–93.18%, 86.16–93.57%, 82.07–91.97%, and 81.67–92.88%, respectively, during 20 batches of AMD applications. For example, in the 20th application, the SO_4_^2−^ ion retention efficiency were 93.59%, 85.71%, 86.16%, 82.63%, and 81.67% in treatments 1, 2, 3, 4, and 5, respectively. The efficiency of retaining Fe ions in calcareous soil was better than that of SO_4_^2−^ ions. The vertical migration of SO_4_^2−^ should be given sufficient attention when AMD contaminates calcareous soil.

Based on Equation 1, the premise of the effective attenuation of SO_4_^2−^ ions during vertical migration of AMD through calcareous soil is mainly that CaCO_3_ reacts with H^+^ ions to produce Ca^2+^, which combines with SO_4_^2−^ ions and is synthesized to CaSO_4_. Komnitsas et al. [39] found that when limestone was used as a neutralizer for AMD treatment, the removal of sulfates from the AMD was promoted by the release of calcium ions due to the dissolution of limestone and the subsequent precipitation of gypsum. However, in the current study, the pH of the filtrate that was collected from the different treatments changed from 6.40 to 7.81 after 20 batches of AMD applications (Figure 2). Therefore, the dissolution capacity of H^+^ on CaCO_3_ was limited when pH was in the range of 6.40–7.81, which may be the main reason for the relatively low SO_4_^2−^ ion retention efficiency in this experiment. Of course, other chemicals (such as MgCO_3_, Al_2_O_3_, and Fe_2_O_3_, etc.) in calcareous soil can also react with H^+^, which further reduces the H^+^ ions concentration which, in turn, further reduces the release of Ca^2+^.

**Figure 4:**
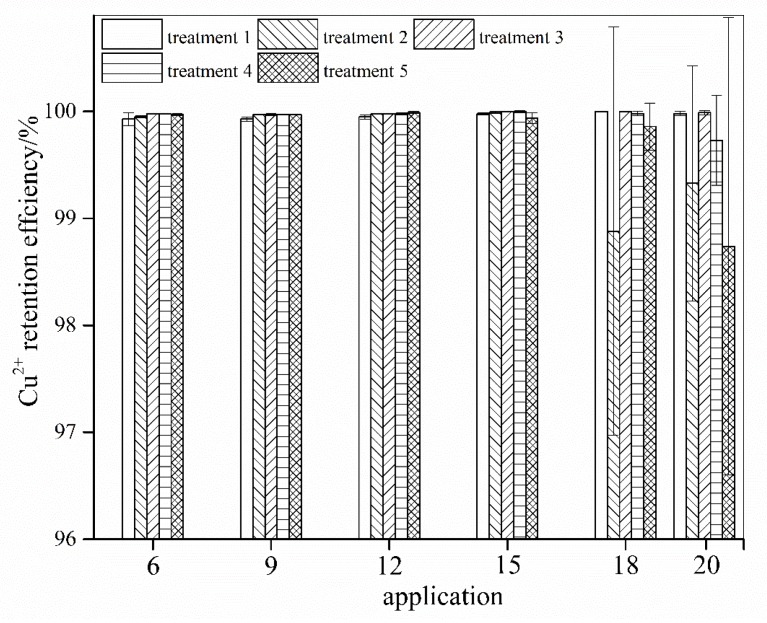
The Cu^2+^ retention efficiency during the addition of simulated acid mine drainage (AMD) into calcareous soil. (Treatments 1, 2, 3, 4, and 5 represent the addition of 22, 44, 66, 88, and 110 mL, respectively of simulated AMD into the soil columns at each application).

AMD is characterized by a low pH, high concentrations of SO_4_^2−^ ions and total Fe, and various concentrations of different metals such as Cu, Al, and Ni. The retention efficiency of calcareous soil for heavy metals was investigated using Cu as the representative pollutant. The Cu retention efficiency in the different treatments at applications 6, 9, 12, 15, 18, and 20 are shown in Figure 4.

Except in treatment 5 (110 mL AMD/application) at application 20, Cu^2+^ was almost completely retained (retention efficiency above ~99%) by the soil in all of the treatments and all of the AMD applications within the treatments. The Cu^2+^ concentration reached 0.62 mg/L (retention efficiency was 98.7%) in the filtrate (pH 6.40) that was collected from the soil columns in treatment 5 (110 mL AMD/application; pH 2.39; Cu^2+^ concentration: 49.27 mg/L) at application 20. According to Britton (1956) [38], Cu hydroxide precipitates at pH ~5.3. Gitari et al. [40] examined fly ash-treated AMD and observed that >75% of the total Cu precipitated out of the solution when the system pH > 5.5. In addition, ferrihydrite or schwertmannite that is produced from Fe^3+^ hydrolysis when AMD contaminates calcareous soil can adsorb Cu^2+^ [41,42], and the organic matter in calcareous soil can complex Cu^2+^ [43]. However, the Cu^2+^ that precipitated at a high pH is probably the main reason for the efficient retention of Cu^2+^ by the soil in this study.

### 3.3. Distribution of H^+^, Fe, S, Cu, and Ca in AMD-Polluted Calcareous Soil

**Figure 5:**
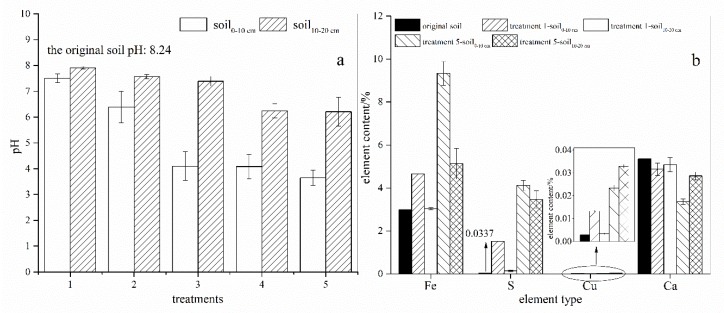
(**a**) The pH, (**b**) Fe, S, Cu, and Ca distributed in the calcareous soil (0–10 cm and 10–20 cm depths) that was contaminated by simulated acid mine drainage (AMD). (Treatments 1 and 5 represent the addition of 22 and 110 mL, respectively, of simulated AMD to the soil columns at each application).

To better investigate the migration behavior in calcareous soil of typical pollutants (H^+^, Fe, S, and Cu) from AMD, as well as a characteristic element (Ca), the soil columns in treatments 1–5 were separated at the middle of each column after 20 batches of applications of AMD and were analyzed. The pH (Figure 5a) of the soil_0__–10cm_ samples was lower than the pH of the soil_10__–20cm_ samples in all of the treatments. Compared with the initial soil pH (8.24), the pH of the soil_0__–10cm_/soil_10__–20cm_ samples in treatments 1–5 were 7.51/7.91, 6.39/7.57, 4.10/7.39, 4.08/6.24, and 3.65/6.21, respectively. There was a significant negative correlation between the amount of AMD added and the pH of the soil_0__–10cm_ samples in all of the treatments (*R^2^* = 0.868) as well as the pH of the soil_10__–20cm_ samples in all of the treatments (*R*^2^ = 0.900).

The distribution of H^+^ at different locations can be indirectly represented by the pH value. In treatment 1, the pH of the soil changed from 8.24 to 7.51 in the soil_0__–10cm_ sample and from 8.24 to 7.91 in the soil_10__–20cm_ sample. Therefore, the distribution of H^+^ ions in the soil_0__–10cm_ sample was 3.84 times that of the soil_10__–20cm_ sample. Similarly, the distribution of H^+^ ions in the soil_0__–10cm_ samples was 18.98, 2270.41, 145.99, and 366.49 times that in the soil_10__–20cm_ samples in treatments 2, 3, 4, and 5, respectively. Notably, the retention efficiency for H^+^ ions in the soil_0__–10cm_ samples reached 2270.41 times more than in the soil_10__–20cm_ samples in treatment 3 (66 mL AMD/application). Yang et al. [5] studied the variation of pH in paddy red soil profiles along the Hengshi River that have been polluted by AMD from the Dabaoshan mining area in South China and found that the pH slightly increased from 4.75 in the soil at a 0–10 cm depth, and to 5.00 in the soil at a 10–20 cm depth. Thus, compared to that in the red soil, the interception of H^+^ in calcareous soil exhibits more distinct “layering”.

The Fe, S, Cu, and Ca concentrations in the soil_0__–10cm_ and soil_10__–20cm_ samples from treatment 1 and treatment 5 were analyzed and the results are shown in Figure 5b. The concentration of Fe in the original soil was 2.99%. After 20 batches of applications of simulated AMD, the concentrations of Fe in the soil_0__–10cm_ and soil_10__–20cm_ samples were 4.65% and 3.03%, respectively (treatment 1), and 9.33% and 5.13%, respectively (treatment 5). Thus, in treatment 1, 97.6% of the total Fe was distributed in the soil_0__–10cm_ sample and 2.4% was in the soil_10__–20cm_ sample. However, in treatment 5, 74.8% of the total Fe was distributed in the soil_0__–10cm_ sample and 25.2% in the soil_10__–20cm_ sample. These results indicated that the distribution of Fe in calcareous soil gradually moved downward through the soil profile as the volume of AMD in the batches of the applications increased.

In treatment 1, 93.1% of the elemental S was distributed in the soil_0__–10cm_ sample and 6.9% was in the soil_10__–20cm_ sample. In treatment 5, the corresponding proportions were 54.3% and 45.7%. These results support the conclusion that compared with elemental Fe, elemental S is more likely to move vertically in calcareous soil that has been contaminated by AMD. Fe ions can precipitate rapidly (almost immediately) and prevent the further downward migration of Fe. However, the migration of S is affected by CaCO_3_ dissolution and CaSO_4_ synthesis, which may not occur at the same locations in the soil profile when AMD infiltrates into the soil. When CaCO_3_ dissolves under the influence of H^+^ ions, Ca^2+^ migrates downward and combines with SO_4_^2−^ during the migration process, which causes elemental S to move further downward than Fe in calcareous soil that has been contaminated by AMD. Yang et al. [5] found that SO_4_^2−^ can react with Fe oxides/hydroxides under acidic conditions in red paddy soil. In the current study of calcareous soil, the SO_4_^2−^ that was retained by Fe oxides/hydroxides was not significant because the elemental S and elemental Fe migration rates were not similar.

In treatment 1, 95.4% of the elemental Cu was distributed in the soil_0__–10cm_ sample (pH 7.51) and 4.6% was in the soil_10__–20cm_ sample (pH 7.91) after 20 applications of simulated AMD. In treatment 5, 40.6% of the elemental Cu was distributed in the soil_0__–10cm_ sample (pH 3.65) and 59.4% was in the soil_10__–20cm_ sample (pH 6.21) after 20 batches of applications of AMD. Cu can form hydroxide precipitates at pH ~5.3 [38]. Therefore, the retention capacity of Cu was reduced in the soil_0__–10cm_ sample in treatment 5 because the 20 batches of applications of large doses of AMD (110 mL/application) caused the pH to decrease to 3.65. Thus, as shown in previous studies, soil pH has a great influence on the migration of elemental Cu.

Elemental Ca is a characteristic element in calcareous soil. In treatment 1, compared with that in the original soil, the Ca content in the soil_0__–10cm_ sample decreased by 8.97% and that of the soil_10__–20cm_ sample decreased by 5.05%. Comparable changes in treatment 5 were decreases of 37.2% and 14.8% in the soil_0__–10cm_ and soil_10__–20cm_ samples, respectively. Notably, the decrease in the Ca content may have been caused by the leaching of Ca^2+^ or by the conversion of CaCO_3_ to CaSO_4_ (and subsequent leaching) when the calcareous soil was subjected to the simulated AMD. For example, the Ca content decreases by 26.5% when 1 unit of CaCO_3_ is transformed to CaSO_4_. Therefore, the Ca in the soil_0__–10cm_ sample in treatment 5 may have been removed by leaching because the Ca content in this soil layer decreased by 37.2%. This phenomenon provides corroborating evidence for the elemental S migration behavior in soil. In other words, in calcareous soil that has been affected by AMD, elemental Ca can enter the lower soil layer from the surface by leaching and binding with SO_4_^2−^ in the lower layer. This interaction forms CaSO_4_, which makes the vertical migration rate of elemental S significantly higher than that of elemental Fe.

### 3.4. Calcareous Soil Mineral Phase before and after AMD Contamination

**Figure 6:**
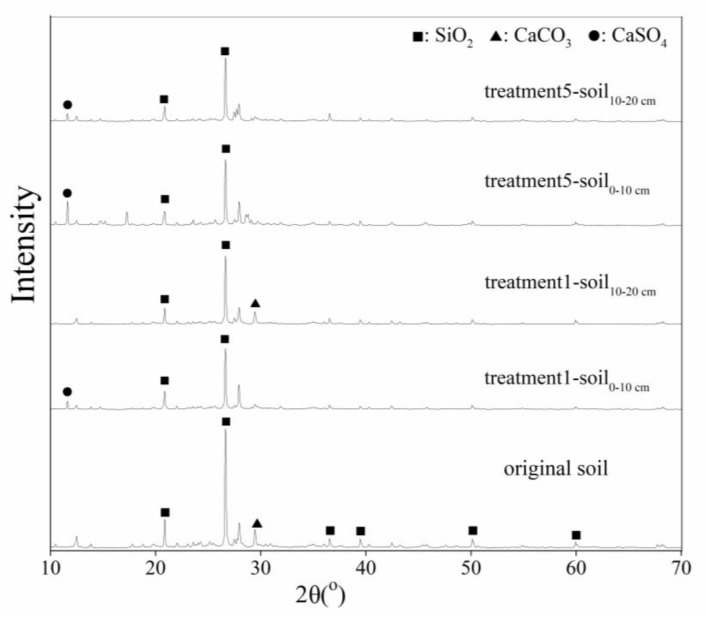
X-ray diffraction patterns for calcareous soil before (original soil) and after the addition of simulated acid mine drainage (AMD). (Treatments 1 and 5 represent the addition of 22 and 110 mL, respectively, of simulated AMD to the soil columns at each application. The soil samples are from 0–10 cm to 10–20 cm depths).

The mineral phase of inorganic soil particles can be explored using XRD technology [44]. In this study, the XRD patterns of the original soil and the AMD-polluted soil were examined. The soil_0__–10cm_ and soil_10__–20cm_ samples from treatments 1 and 5 were used as examples and are shown in Figure 6. According to the Joint Committee on Power Diffraction Standards data files [45] cards No. 46-1045 and 47-1743, the patterns in Figure 6 indicate that the dominant substances in the original soil were SiO_2_ (characteristic peak in XRD patterns at 2*θ* = 26.64° and 20.86°) and CaCO_3_ (calcite, characteristic peak in XRD patterns at 2*θ* = 29.40°).

Although the original soil also contained other oxides (Fe_2_O_3_, Al_2_O_3_, etc.), organic matter, and other material, the characteristic diffraction peaks of these substance did not exhibit an obvious XRD pattern because the SiO_2_ and CaCO_3_ accounted for such a large proportion of the calcareous soil [46,47]. After the calcareous soil was subjected to AMD, the characteristic peak (2*θ* = 29.40°) of CaCO_3_ could not be observed in the soil_0__–10cm_ samples from either treatment 1 or treatment 5, nor in the soil_10__–20cm_ sample from treatment 5. By comparing the XRD patterns of these treatments to JCPDS card (No. 33-0311) and the results from previous research, an interesting peak (2*θ* = 11.60°) characteristic of CaSO_4_·2 H_2_O was observed in the soil_0__–10cm_ samples from treatments 1 and 5 and in the soil_10__–20cm_ sample from treatment 5. This result provided great support for the retention capacity of soil for elemental S, as shown in Figure 5. Notably, the characteristic peak (2*θ* = 11.60°) of CaSO_4_·2H_2_O was not observed in the XRD pattern for the soil_10__–20cm_ sample from treatment 1 because elemental S barely accumulated in this soil layer. Moreover, the XRD patterns that are shown in Figure 6 directly indicate that the transformation of CaCO_3_ to CaSO_4_ did actually occur in the calcareous soil that was contaminated by AMD.

**Figure 7:**
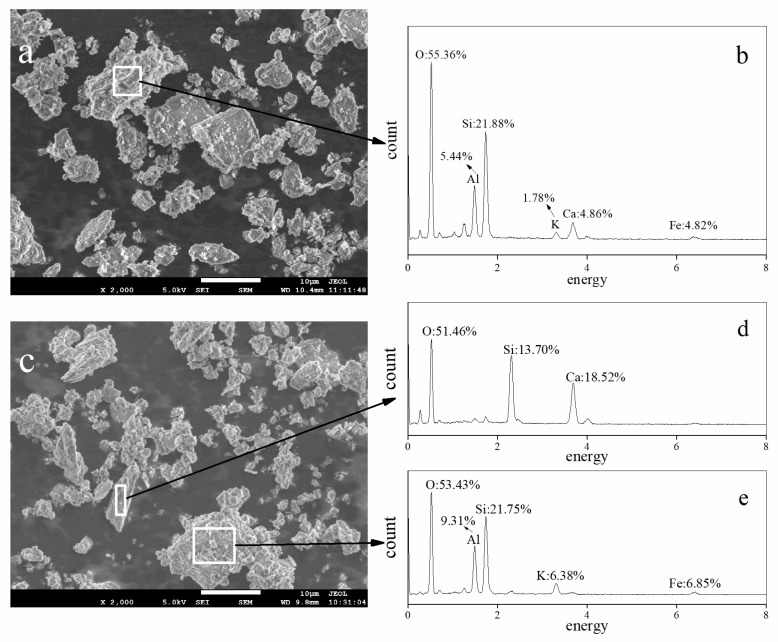
Scanning electron microscopy (SEM) images and energy dispersive X-ray spectroscopy (EDS) spectra of the calcareous soil before and after the addition of simulated acid mine drainage (AMD). (**a**) SEM of the original soil; (**b**) EDS of the particle marked with a square in 7 (**a**); (**c**) SEM of soil_0__–10cm_ in treatment 5 (110 mL AMD/application); (**d**) EDS of the particle marked with a narrow rectangle in 7 (**c**); (**e**) EDS of the particle marked with a square in 7 (**c**).

SEM images of calcareous soil before and after being subjected to AMD are shown in Figure 7. The SEM image of the original calcareous soil (Figure 7a) shows that the mineral morphology of the calcareous soil was a mixture of crystal and amorphous materials. The energy dispersive X-ray spectroscopy (EDS) spectrum of the representative particle that is identified in Figure 7a showed that the main elements in the original calcareous soil were O (55.36 wt%), Si (21.88 wt%), Al (5.44 wt%), Ca (4.86 wt%), Fe (4.82 wt%), and K (1.78 wt%) (Figure 7b). These data suggest that the main chemical components in the original soil were SiO_2_, Al_2_O_3_, CaCO_3_, Fe_2_O_3_, K_2_O, etc. However, the SEM image of the AMD-contaminated soil (Figure 7c) shows the rod-shaped morphology of CaSO_4_·2H_2_O in the soil_0__–10cm_ sample from treatment 5. Furthermore, the chemical composition of CaSO_4_·2H_2_O was confirmed by the EDS patterns that are shown in Figure 7d. Of course, the elements Si, Al, Fe, and K could also exist in other particles in the AMD-contaminated soil (Figure 7e).

## 4. Conclusions and Prospects

Shanxi, a province in the northern part of China, is rich in mineral resources, and AMD is a typical contaminant in mining areas in Shanxi. The dominant type of soil in Shanxi is calcareous soil. To our knowledge, this study is the first to address the migration behavior of AMD pollutants, such as H^+^, Fe, S, and heavy metals (using Cu as an example) in calcareous soil in the Shanxi area. The results described in Section 3 support the following conclusions: Calcareous soil has a great pH buffering effect on AMD. This effect is significantly and negatively correlated with the amount of AMD added, as is indicated by pH changes in the top 0–20 cm layer of the soil. In the calcareous soil that is affected by AMD, CaCO_3_ is transformed into CaSO_4_. Almost all Fe ions from AMD can be retained in the 0–20 cm surface soil and more than 80% of SO_4_^2−^ can be retained in this layer. Thus, the retention efficiency of calcareous soil is greater for Fe than for SO_4_^2−^. The vertical migration of elemental S in calcareous soil is obviously greater than that of elemental Fe. Elemental Cu, a representative of other heavy metals that are often contained in AMD, can be totally retained by calcareous soil in some conditions. However, the Cu has a tendency to migrate downward with the gradual acidification of the upper soil profile.

The outcomes of this study are valuable for understanding the pollution of calcareous soil by AMD. Calcareous soil had a strong buffer effect on AMD acidity due to it containing a large amount of calcium carbonate, which resulted in a large number of AMD pollutants’ (Fe, S, and heavy metals) vertical migration slowly, and mainly accumulated on the surface (0–20 cm) of the soil. However, the vertical migration of sulfates is faster than that of iron, which increases the possibility of groundwater sulfate-contamination. The removal of sulfate from AMD is our team’s main future research direction.
